# Supplementary material for: Wastewater Analyses for Psychoactive Substances at Music Festivals: A Systematic Review
Source: Behav Sci (Basel). 2025 Dec 3;15(12):1672. doi: 10.3390/bs15121672 (PMC12729779; doi:10.3390/bs15121672)
Supplement: Supplementary file 1 [file behavsci-15-01672-s001.zip › behavsci-3880742-supplementary.pdf]

Supplementary Materials:

Table S1: PRISMA 2020 Checklist.

	Item #	Checklist item	Location where item is reported
TITLE			
Title	1	Identify the report as a systematic review.	Title; page 1.
ABSTRACT			
Abstract	2	See the PRISMA 2020 for Abstracts checklist.	Abstract; page 1.
INTRODUCTION			
Rationale	3	Describe the rationale for the review in the context of existing knowledge.	Sections 1.1. to 1.4.; pages 1 to 3.
Objectives	4	Provide an explicit statement of the objective(s) or question(s) the review addresses.	Section 1.5. "Aim"; page 3.
METHODS			
Eligibility criteria	5	Specify the inclusion and exclusion criteria for the review and how studies were grouped for the syntheses.	Section 2.2. "Inclusion and exclusion criteria"; page 4.
Information sources	6	Specify all databases, registers, websites, organisations, reference lists and other sources searched or consulted to identify studies. Specify the date when each source was last searched or consulted.	For the registration see section 2; page 4. For the sources see section 2.1. "Search strategy"; page 4.
Search strategy	7	Present the full search strategies for all databases, registers and websites, including any filters and limits used.	Section 2.1. "Search strategy"; pages 4.
Selection process	8	Specify the methods used to decide whether a study met the inclusion criteria of the review, including how many reviewers screened each record and each report retrieved, whether they worked independently, and if applicable, details of automation tools used in the process.	Section 2.3. "Screening / Selection Process"; page 5; for details of automation tools used see Section 2.1. "Search strategy"; page 4.
Data collection process	9	Specify the methods used to collect data from reports, including how many reviewers collected data from each report, whether they worked independently, any processes for obtaining or confirming data from study investigators, and if applicable, details of automation tools used in the process.	Section 2.3. "Screening / Selection Process"; page 5; for details of automation tools used see Section 2.1. "Search strategy"; page 4.
Data items	10a	List and define all outcomes for which data were sought. Specify whether all results that were compatible with each outcome domain in each study were sought (e.g. for all measures, time points, analyses), and if not, the methods used to decide which results to collect.	Section 2.4. "Data extraction"; page 5.
	10b	List and define all other variables for which data were sought (e.g. participant and	Section 2.4. "Data extraction"; page 5.

 https://doi.org/10.3390/bs15121672

	Item #	Checklist item	Location where item is reported
		intervention characteristics, funding sources). Describe any assumptions made about any missing or unclear information.	
Study risk of bias assessment	11	Specify the methods used to assess risk of bias in the included studies, including details of the tool(s) used, how many reviewers assessed each study and whether they worked independently, and if applicable, details of automation tools used in the process.	Section 2.3. "Screening / Selection Process"; page 5. For details see supplementary table S2; questions 3 to 7 of the JBI Critical Appraisal Tool specify information pertaining to the risk of bias.
Effect measures	12	Specify for each outcome the effect measure(s) (e.g. risk ratio, mean difference) used in the synthesis or presentation of results.	Effect measures such as risk ratios and mean differences for each outcome were not assessed; see section 4.2. "Limitations"; pages 29 to 33.
Synthesis methods	13a	Describe the processes used to decide which studies were eligible for each synthesis (e.g. tabulating the study intervention characteristics and comparing against the planned groups for each synthesis (item #5)).	Section 2.6. "Analysis"; page 5 and Table 1; pages 7 to 16.
	13b	Describe any methods required to prepare the data for presentation or synthesis, such as handling of missing summary statistics, or data conversions.	Section 2.4. "Data extraction"; page 5. Section 2.6. "Analysis"; page 5.
	13c	Describe any methods used to tabulate or visually display results of individual studies and syntheses.	Section 2.4. "Data extraction"; page 5. Table 1; pages 7 to 16
	13d	Describe any methods used to synthesize results and provide a rationale for the choice(s). If meta-analysis was performed, describe the model(s), method(s) to identify the presence and extent of statistical heterogeneity, and software package(s) used.	Section 2.6. "Analysis"; page 5.
	13e	Describe any methods used to explore possible causes of heterogeneity among study results (e.g. subgroup analysis, meta-regression).	See section 4.2.9. "Causes of heterogeneity"; page 31.
	13f	Describe any sensitivity analyses conducted to assess robustness of the synthesized results.	We were not able to perform sensitivity analyses; see section 4.2.9. "Causes of heterogeneity"; page 31.
Reporting bias assessment	14	Describe any methods used to assess risk of bias due to missing results in a synthesis (arising from reporting biases).	We used the Joanna Briggs Institute (JBI) Critical Appraisal Tools for Analytical Cross. Sectional Studies (Joanna Briggs Institute, 2025); see supplementary Table S2.
Certainty assessment	15	Describe any methods used to assess certainty (or confidence) in the body of evidence for an outcome.	We used the Joanna Briggs Institute (JBI) Critical Appraisal Tools for Analytical Cross Sectional Studies (Joanna Briggs Institute, 2025); see supplementary Table S2.

	Item #	Checklist item	Location where item is reported
RESULTS			
Study selection	16a	Describe the results of the search and selection process, from the number of records identified in the search to the number of studies included in the review, ideally using a flow diagram.	Section 3.1. "Search results"; page 6 and PRISMA Flow Diagram; page 6.
	16b	Cite studies that might appear to meet the inclusion criteria, but which were excluded, and explain why they were excluded.	PRISMA Flow Diagram; page 6.
Study characteristics	17	Cite each included study and present its characteristics.	Section 3.2. "Study Characteristics"; page 6 and Table 1; pages 7 to 14.
Risk of bias in studies	18	Present assessments of risk of bias for each included study.	We used the Joanna Briggs Institute (JBI) Critical Appraisal Tools for Analytical Cross Sectional Studies (Joanna Briggs Institute, 2025); see supplementary Table S2. See also section 4.2.8. "Risk of bias"; page 31.
Results of individual studies	19	For all outcomes, present, for each study: (a) summary statistics for each group (where appropriate) and (b) an effect estimate and its precision (e.g. confidence/credible interval), ideally using structured tables or plots.	Festival and descriptive sample details are reported in table 1 and on pages 15 to 24.
Results of syntheses	20a	For each synthesis, briefly summarise the characteristics and risk of bias among contributing studies.	See section 4.2.8. "Risk of bias"; page 31. For gender bias and bias relating to sampling and technical-analytical constraints see sections 4.2.3., 4.2.4., and 4.2.5.
	20b	Present results of all statistical syntheses conducted. If meta-analysis was done, present for each the summary estimate and its precision (e.g. confidence/credible interval) and measures of statistical heterogeneity. If comparing groups, describe the direction of the effect.	A statistical synthesis was not performed.
	20c	Present results of all investigations of possible causes of heterogeneity among study results.	Section 4.2.9. page 32.
	20d	Present results of all sensitivity analyses conducted to assess the robustness of the synthesized results.	Given the nature of the data obtained, a sensitivity analysis to assess the robustness of the synthesized results was not deemed meaningful.; see section 4.2.9. "Causes of heterogeneity"; page 31.
Reporting biases	21	Present assessments of risk of bias due to missing results (arising from reporting biases) for each synthesis assessed.	In our systematic review, missing results pertains to drugs that were not measured. This is covered in section 4.2.8. "Risk of bias" under "detection bias"; page 31.
Certainty of evidence	22	Present assessments of certainty (or confidence) in the body of evidence for each outcome assessed.	Table 1; pages 7 to 14. Section 4.2. "Limitations"; pages 28 to 32.

	Item #	Checklist item	Location where item is reported
DISCUSSION			
Discussion	23a	Provide a general interpretation of the results in the context of other evidence.	Section 4.1. "Summary of Results", pages 24 to 28.
	23b	Discuss any limitations of the evidence included in the review.	Section 4.2. "Limitations"; pages 28 to 32.
	23c	Discuss any limitations of the review processes used.	Section 4.2.1. "Limitations of this systematic review"; pages 28 to 32.
	23d	Discuss implications of the results for practice, policy, and future research.	Section 4.4. "Implications and Future Directions"; pages 31 and 32. Section 5. "Conclusions"; pages 32 and 33.
OTHER INFORMATION			
Registration and protocol	24a	Provide registration information for the review, including register name and registration number, or state that the review was not registered.	The review was registered with the Open Science Framework (OSF; https://osf.io/9aq7p/). See section 2; page 4.
	24b	Indicate where the review protocol can be accessed, or state that a protocol was not prepared.	The protocol can be accessed from https://osf.io/9aq7p/
	24c	Describe and explain any amendments to information provided at registration or in the protocol.	See above. No amendments were made to the initial protocol.
Support	25	Describe sources of financial or non-financial support for the review, and the role of the funders or sponsors in the review.	See funding statement on page 34.
Competing interests	26	Declare any competing interests of review authors.	See conflicts of interest statement on page 34.
Availability of data, code and other materials	27	Report which of the following are publicly available and where they can be found: template data collection forms; data extracted from included studies; data used for all analyses; analytic code; any other materials used in the review.	The exact search strategy for PubMed, Embase, and MEDLINE (via Ovid) is given in section 2.1 "Search strategy" on page 4. All extracted data are reported in Table 1 on pages 7 to 14 (with direct links) and in the Supplementary Material.

Table S2: Quality assessment of included studies using the Joanna Briggs Institute (JBI) Critical Appraisal Tool for Analytical Cross Sectional Studies (Joanna Briggs Institute, 2025). Questions 3 to 7 cover information pertaining to the risk of bias.

Study	Were the criteria for inclusion in the sample clearly defined?	Were the study subjects and the setting described in detail?	Was the exposure measured in a valid and reliable way?	Were objective, standard criteria used for measurement of the condition?	Were confounding factors identified?	Were strategies to deal with confounding factors stated?	Were the outcomes measured in a valid and reliable way?	Was appropriate statistical analysis used?
Bade et al., 2024	Yes	No	Yes	Yes	Unclear	No	Yes	N/A

Bengalia et al., 2020	Yes	Yes	Yes	Yes	Yes	Yes	Yes	Yes
Berset et al., 2010	Yes	Yes	Yes	Yes	Yes	Yes	Yes	Unclear
Bijlsma et al., 2014	Yes	Yes	Yes	Yes	Yes	Yes	Yes	Unclear
Bijlsma et al., 2020	Yes	Yes	Yes	Yes	Yes	Yes	Yes	Unclear
Bodik et al., 2016	Yes	Yes	Yes	Yes	Yes	Yes	Yes	Unclear
Bran-deburova et al., 2020	Yes	Yes	Yes	Yes	Yes	Yes	Yes	Unclear
Brett et al., 2021	Yes	Yes	Yes	Yes	Yes	No	Yes	Unclear
Devault et al., 2020	Yes	Yes	Yes	Yes	Yes	Unclear	Yes	Yes
Geuens et al., 2022	Yes	Yes	Yes	Yes	Yes	Unclear	Yes	Unclear
Gjerde et al., 2019	Yes	Yes	Yes	Yes	No	No	Yes	Yes
Gomes et al., 2024	Yes	Yes	Yes	Yes	Yes	No	Yes	Unclear
Hoegberg et al., 2017	Yes	Yes	Yes	Yes	Yes	No	Yes	No
Jiang et al., 2015	No	Yes	Yes	Yes	Yes	No	Yes	Yes
Kinyua et al., 2016	Yes	Yes	Yes	Yes	Yes	No	Yes	No
Lai et al., 2013	Yes	Yes	Yes	Yes	Yes	Unclear	Yes	Yes
Maasz et al., 2021	Yes	Yes	Yes	Yes	Yes	Unclear	Yes	Unclear
Mackulak et al., 2014	Yes	Yes	Yes	Yes	Unclear	Unclear	Yes	Unclear
Mackulak et al., 2018	Yes	Yes	Yes	Yes	Yes	Yes	Yes	Yes

Nadarajan et al., 2024	Yes	No	Yes	Yes	Yes	Unclear	Yes	Yes
Puljevic et al., 2024	Yes	No	Yes	Yes	Yes	Unclear	Yes	Yes
Senta et al., 2023	Yes	Yes	Yes	Yes	Yes	Yes	Yes	Yes
Sutlovic et al., 2021	Yes	Yes	Yes	Yes	Unclear	Unclear	Yes	Unclear
